# Mental health consequences of home demolition policy towered Palestinians: Literature review

**DOI:** 10.1186/s13033-021-00472-0

**Published:** 2021-05-29

**Authors:** Mohammed Marie, Sana SaadAdeen

**Affiliations:** 1 Faculty of Medicine and Health Sciences-Nablus, AN-Najah National University, Nablus, Palestine; 2 Primary health care centers-Ministry of Health-Palestine, Nablus, Palestine; 3 Community Mental Health Nursing program, An-Najah National University, Nablus, Palestine

**Keywords:** Home demolition, Mental health, Palestine, Gaza strip, West Bank

## Abstract

**Abstract:**

Home demolition is considered a miserable and frequent reality of life for thousands of Palestinian people. Recently, in January 2020, at least 44 structures were demolished by Israeli forces, displacing at least 87 people. Studies have revealed high levels of stress was associated with losing a family home during home demolition, and the stress doesn’t only carry out immediate effects but also long-term mental consequences. This paper aims to provide an overview of the literature and established studies related to the mental health consequences of home demolitions in Palestine.

**Methods:**

PubMed, Science Direct, Semantic Scholar and Google Scholar were used to search for materials for the critical analysis of empirical articles. The following aspects were taken into consideration: study type, sample, and key findings.

**Results:**

In this review, nine studies related to mental health consequences of home demolition were found and considered in this paper. The results indicate depression, stress, anxiety, phobias and lack of hope in the future are mutual themes running throughout the lives of those Palestinian families who have actually experienced home demolition along with those who have experienced a constant threat of demolition to their homes. In addition, many of life stressors such as lack of educational opportunities, low incomes, and a tendency to live in poor housing conditions after and before demolition also play a role in developing serious mental disorders.

**Conclusion:**

As primary prevention, the practice of home demolition in Palestine has to be ended. In addition to conduct a constant monitoring of the resulting displacement and the damage caused, and ensuring the necessary assistance in particular mental health and psychological support for victims of home demolitions.

**Supplementary Information:**

The online version contains supplementary material available at 10.1186/s13033-021-00472-0.

## Background

Palestine (Occupied Palestinian Territories) is an eastern Mediterranean country that seeks independence and freedom. The term "oPt" as a whole refers to the geographic region of the Palestinian territory occupied by Israel and in which this small region has attracted the world’s attention for a long time [1]. Before the World War II, Palestine was under British mandate. In 1948, Palestine name was removed and replaced with a new nation (Israel). Britain was committed to support the established Zionist movement that sought to create “a national home for the Jewish people” in Palestine [2]. While the Israelis considered it "liberation," the Palestinians considered the beginning of the "catastrophe" ‘Nakba’ [3]. The violent birth of Israel led to a major displacement of the Arab population. According to the Palestinian Central Bureau of Statistics in the year 2012, 7.4 million (66%) of the global population of 11.2 million Palestinians have been displaced forcibly from their homeland [4]. As a result, most of Palestinian people were evicted forcibly, displaced from their homes and became refugees into different neighborhood countries e.g. Jordan and Syria. The others remained in the occupied Palestine [5].

In 2002, Israel started to build a physical barrier with parts of it isolating the Palestinians’ cities and villages. Israel called it a ‘fence’ and Palestinians called it a ‘Separation Wall’ [6]. In the years 2008, 2012 and 2014, there was a prolonged siege that included movement restrictions on food and individuals, especially in the Gaza Strip [7]. One of the many ways in which the Israeli government controls the Palestinian population–alongside theses: Separation Wall, water shortages, the appropriation of Palestinian land and the segregation of Palestinians living in different occupied areas–is through home demolition [8]. Home has such a central meaning in Palestinian culture. It is not only a shelter, but also the heart of family life [9]. Inside every Palestinian home there are mutual memories of pain and joy as well as attachment to familiar objects. Palestinians believe in the security of home since they consider being in place, as part of a family, and as a resident in their village and thus part of a broader nation. Moreover, the idea of security has a historical meaning, and this means that homes would be built for the express purpose of repelling invaders [10].

The urban architecture of Palestine prior to 1850 was relatively elegant [11]. Two types of house predominated in Palestine from the second millennium BCE through to the modern era: the simple house found commonly in rural areas and the courtyard house found mostly in urban centers [11, 12]. The sense of "rootedness" and "unmediated connectedness" which characterized Palestinian Arab architecture was admired by many travelers [13]. The majority of Palestinians live in the same house and neighborhood their entire life, and moving to another house or neighborhood is not done regularly because in many cases they maintain strong social ties with their families and neighbors [10, 14]. Although the behaviors of races and cultures differ among people around the world, the basic needs they satisfy are very similar. According to the psychologist Abraham Maslow, the need for shelter is one of the first and most important human needs [15]. In any given year approximately 750 000 people lose their housing through demolition worldwide. In many instances, those living in locations targeted by demolition have little say in the disposition of their neighborhood and often face difficulty in finding appropriate replacement housing [16]. Although, thousands of Palestinian populations throughout the Occupied Palestinian Territories (oPt) and (Palestinians who are living in occupied land since 1948) has received humanitarian response to such practices through shelters, they were subject to the continuity of demolition or confiscation [17]. According to Amnesty International reports, it highlighted Israeli missile attacks on the Gaza Strip, which destroyed entire families. Most of their homes were crowded with relatives, and they were attacked by large missals. For example, the organization examined eight cases, in which Israeli attacks targeting at least 111 people were killed and civilian homes destroyed. Survivors described the terrible destruction of their homes as they searched the bodies of children and loved ones and most of the West Bank residents were very concerned about their relatives in the Gaza Strip during the military operation [18, 19].

The WHO has deemed housing as a preexisting condition of health, since it is a fundamental human right and a major social determinant of health [20, 21]. Worldwide, approximately 60% of the population have severe and disabling mental health disorders, which are expected to rise to 75% after severe trauma or loss [22]. According to WHO, an increase in the burden of mental and psycho-social disorders can be expected in a population experiencing prolonged occupation, lack of personal security, severe movement restrictions and human rights violations, including displacement in a post-conflict situation” [23]. In general, silent ethnic cleansing practices in Palestine in the past seventy years, the impact on mental health is one of the most important consequences for the Palestinian population [24]. Such of policy of house demolitions, along with settlements, restricted movement, exposure to threats or even killed, have left thousands of Palestinians subject to severe distress which could lead to major psychological and mental disorders [19].

According to the WHO, mental health challenges” is "one of the most important public health challenges" in the OPT [25]. In general, there are many factors, including economic, political, social and cultural factors that play a role in determining the state of mental health and providing services to residents [26]. It is well established that there is a gap between the mental health needs of the Palestinian population and the provision of mental health services. For instance, the mental health system faces specific challenges related to occupation and political conflict. According to Marie et al. [27] restrictions on freedom and movement greatly limit patients receiving care outside their area of residence, in addition to the cost of treatment, the inconsistent availability of medications on the WHO Essential Medicines List, as well as insufficient specialists and by absence of interdisciplinary teamwork.

Indeed, home demolitions can lead to multi-faceted negative consequences: family disruptions, loss of culture, loss of identity, mental health problems, et cetera. Given these challenges in light of policy of home demolitions, there is a pressing need to look around mental health condition for whom experienced their homes demolished. This paper aims to provide an overview of the literature and established studies related to the mental health consequences of home demolitions for Palestinian families who have actually experienced home demolition along with those who have experienced a constant threat of demolition to their homes.

### Israeli home demolition policy

The roots of Israel's house demolition policy date back to British Mandate, which has always been the overwhelming weapon of colonialism. The home demolition policy was implemented under Article 119 of the "Defense of Law (Emergency)" regulations. Although the British claimed to repeal the law upon their departure, the Israeli regime continued to use it [28]. The military government implements demolitions by either detonating the house with explosives or destroying it with bulldozers. When demolitions seem unattainable for technical reasons, the military government usually closes houses and closes their doors and windows using bricks or metal sheets. In cases of destruction and sealing, families are forbidden to rebuild their homes or use their lands in any way [29]. For instance, during the first year of intifada in 1987, 125 houses were demolished and 41 were sealed; in the second year, 158 houses were demolished and 80 were sealed; in the third year, 88 houses were demolished and 96 were sealed; and in the fourth year, 47 houses were demolished and 48 were sealed. In the fifth year (beginning December 1991) 8 houses were demolished and 22 were sealed [30].

Since the beginning of the second intifada in September 2000, the rate of house demolitions has risen significantly. According to the UN Office for Co-ordination of Humanitarian Affairs (UNOCHA) which monitors and maps house demolitions, East Jerusalem is experiencing an increasing number of demolitions and population displacement. In the ten years between 2004 and 2014, 516 housing units were demolished in East Jerusalem; 59 were carried out by the home owners to save the amount that Israel demands for demolition. As a result, more than 2000 Palestinians people have been made homeless. The vast majority of these homes belonged to Palestinians [5]. The Destruction and confiscation of Palestinian houses in East Jerusalem by the Israeli authorities, led to displacement and expulsion of the inhabitants. Men, women, and children were left homeless, mentally scarred, and economically and socially ruined, all because they tried to improve their lives and provide themselves with a place to live [31]. Recently, during the month of June 2019, at least 64 structures were demolished in the occupied Palestinian Territories (including East Jerusalem) by Israeli forces, displacing at least 60 people- including 27 children- and affecting a further 18,774 people [32, 33]. During the month of January 2020, at least 44 structures were demolished in the occupied Palestinian Territories (including East Jerusalem) by Israeli forces, displacing at least 87 people- including 56 children- and affecting a further 205 people. Furthermore, in the Naqab desert, the entire Bedouin village of al-Aragib has been demolished three times during the month of January 2020 alone and more than 173 times all together in the past ten years [34].

On the other hand, the possibility of Palestinian construction is restricted severely. For instance, building is limited to only 13 per cent of East Jerusalem and only one per cent of Area C (which is totally managed by Israeli military forces). Moreover, more than 94% of the 3,750 requests for planning permission made by Palestinians between 2010 and 2012 were rejected by the Israeli authorities. As a result, Palestinians wishing to extend their homes or expand their communities in order to accommodate family and population growth face impossible bureaucratic barriers and have no other option but to build without a military permit [5].

According to Israeli authorities, Palestinian homes are demolished for various reasons: the land they own has been declared by Israel “open green space;” they have no building permit (which Israeli authorities rarely grant to Palestinians); the slope of their land is adjudged as “too steep;” their houses are too near settlements or Israeli only highways (although the houses were there first), out of collective punishment for some action the punished people had nothing to do with, the “clearing” of vast tracts of land for military/security purposes, destruction for the sake of expanding roads, settlements and the “Separation Barriers;” houses “cleared” to make passage safe for settlers or for other security purposes, homes representing “collateral damage,” and more of unjustified causes [35].

Today, tens of thousands of Palestinians carry demolition orders with their hands and live-in fear, and they wonder when bulldozers will arrive to demolish their homes. The hidden message of the bulldozers is clear: "Get out." We uprooted you from your homes in 1948 and prevented you from returning, and now we will force you to leave from all of the Land of ‘Israel’ [36].

## Methodology

### Search strategy

The literature review in this article was gathered through searching in electronic databases. The following electronic databases were searched: PubMed, Science Direct, Semantic Scholar and Google Scholar. The keywords used in the searching process are: Home demolition AND Palestine, Home demolition AND Gaza Strip, Home demolition AND West Bank. Mental Health AND Palestine. Mental Health AND Home demolition. These words were also used to search in the Arabic language to identify articles indexed in An-Najah University Journal for Research. Search history and study filtration show in Additional file 2: Table S2. Twenty-five relevant articles and reports were included. Ten articles related to home demolition in West bank and Gaza. Seven reports related to Statistics of home demolitions in Palestine and nine articles related to mental health consequences of home demolition were found and considered in this paper. Additional papers -did not come to light in the electronic database search- were obtained via an examination of reference lists of published papers. The following aspects were considered for critical analysis of these articles: study type, the study instrument, aim, sample, and results. The study was conducted following the Preferred Reporting Items for Systematic Reviews and Meta-Analyses (PRISMA) guidelines shown in Additional file 1. The inconsistencies are resolved by consensus between authors which include the critical appraisal and the writing up of the sections.

### Selection criteria

Due to the lack of mental health studies in Palestine; all of the related articles about mental health consequences of home demolition policy towered Palestinians were included in this study. there were also practical difficulties to access to the studies in libraries shelves in east Jerusalem and the Gaza strip because of the presence of military barriers and the Separation Wall.

A. **Quality assessment:** The quality assessment of each study was assessed according to a checklist. The checklist consists of the following items: including clear study aims, adequate sample size, response rate reported, and losses have given, an adequate description of data, appropriate statistical analysis. a representative sample (with justification), clear inclusion and exclusion criteria, valid and reliable measure of mental health. (SS) investigated independently assessed article quality, and inconsistencies were resolved by the principal investigator (MM).
B. **Characteristics of included studies:** Searching history shown in Additional file 2:Table S1 and the characteristics of the included studies are presented in table Additional file 2:Table S2 (Studies Characteristics were added into two group Quantitative and Qualitative). After reviewing the depth of selected search findings and obtaining necessary data. Nine studies regarding mental health consequences of home demolition policy towered Palestinians, were included in this review conducted in Palestine (West Bank and Gaza Strip). The two reviewers independently charted the data, discussed the results, and continuously updated the data-charting form in an iterative process. We grouped the studies by the topic they studied. For the critical analysis of empirical articles, the following aspects were considered: study type, the survey instrument used, aim, sample, and the key findings. A data-charting form was jointly developed by two reviewers to determine which variables to extract.

## Results

### An overview of mental health consequences related to home demolition in Palestine

Since the beginning of the Al-Aqsa Intifada, many children have experienced several traumatic events. Numerous studies and surveys were conducted on the impact on Palestinian children during and after the crisis. One of these studies aimed to assess the nature and severity of emotional problems among Palestinian children living in Gaza Strip whose homes had been bombarded and demolished. The sample consisted of 180 children: 91 (51%) who had been exposed to bombardment and home demolition, and 89 (49%) controls who had not. The sample completed self-report measures of posttraumatic stress, anxiety, and fears. The results showed children exposed to bombardment and home demolition scored significantly higher on total fear scores than did controls. The most frequently reported items of (post-traumatic stress symptoms occurring much or most of the time) in exposed children were: identifies event as extremely stressful (66%), difficulty in concentrating (58%), sleep disturbance (57%), and avoidance of reminders (52%). The most frequently reported fears of children exposed to home bombardment were: being at a high place, you feel that it will collapse (69 of 91, 76%), feeling scared in a dark place (65, 71%), fears of being in a closed space (63, 69%), fears of height and high buildings (63, 69%), fears of things and people the child knows will not hurt him (61, 67%), and fears of having an untreatable disease (58, 64%) [37].

Studies have shown fear and lack of feeling safe is one of the most causes of mental disorders, such as anxiety, phobias, and depression [38]. Under normal circumstances, families use the physical structure of their home to ensure privacy and safety, opening the door to welcome guests and closing it to refuse entry to unwelcome outsiders. By using five focus groups, a study conducted in 2008 to draw out narratives of 32 Palestinian women who were affected by political violence. Respondents were drawn from general health, children's and eye-care clinics. The groups included the following number of participants: Hebron(5), Nablus(7), Jerusalem(4), Qalqiliya(10), Tulkarem(6). Women described how they mobilized the home for economic, familial and cultural survival. Surveillance, home invasions, and actual or threatened destruction of women's home environments provoked fear, anxiety, grief, humiliation, and helplessness, particularly as women struggled to protect their children. Women's narratives of relentless surveillance highlight their constant doubt regarding the integrity of their homes. One woman said simply, “This is our life,” and other said, “Where is our freedom?”. Under incessant surveillance, home is no longer a refuge of privacy and autonomy but rather a place of fear and insecurity. One woman from Balata refugee camp in Nablus said, “Every night, near 2:00 am, Soldiers start to scream, shoot, and torment our neighborhood. They have entered our house many times for no reason, with no consideration for children or elders creating fear by using forceful [39].

For indigenous people, prolonged residence in one place and attachment to their land are significant elements of collective identities. As a result, forced displacement and threat of home loss may prompt fear of identity loss, and might be related to high stress levels and post-trauma [40]. In addition to, when this spatial identity is interrupted by conflict, communities can become dysfunctional, and this can have serious impacts, including psychological disruption [41]. The Arab Bedouins have lived in the Negev (Naqab), now part of Israel’s southern region, since long before the establishment of the state of Israel in 1948 [42]. Arab Bedouins are among the poorest and most disadvantaged groups in Israel [43]. About half of the Arab Bedouins—some 90,000 people—now live in shacks and other temporary dwellings without access to basic infrastructure [44]. A cross-sectional study was conducted between July 2008 and January 2009 among Arab Bedouin women through 14 Mother and Child Health (MCH) clinics in southern Israel. Their ages were between 18 and 49 years. A total of 464 women agreed to participate in the study and were interviewed by using a structured Arabic-language questionnaire to examine the relationship between risk of home demolition and symptoms of depression (DS). The participants’ women were asked: Is your house threatened with demolition? In addition to the housing characteristics such as the type of building, house crowding, electricity and water supply, and house location: whether the house was located in a legally recognized or unrecognized village (by the Israeli government as legal). Results showed that 27.2% of participants reported that their house is under threat of demolition. Women living in a house under threat of demolition had significantly higher Depression Scale (more than six symptoms a week). Furthermore, these women had poorer education, family source of income, and literacy, compared to those in stable housing. Also, sixty four percent of them reported that their house was not connected to the electrical grid and house crowding was greater than those in stable housing. The Study found threat of housing demolition creates higher Depression Scale, even after adjusting for women’s, physical features of the house, and house location [45].

Overall, studies have shown that displaced persons tend to live in poor housing conditions and to have low security and income, since they lose all of their belongings and social standing when they are displaced [46]. Several Non-governmental organizations NGOs have reported about the consequences of home demolition has on the victims. For example, Save the Children UK in 2009, completed a research study on the pressures and impacts associated with forced displacement in the occupied Palestinian territory. The research aimed to investigate the striking vulnerabilities of families living in high-risk areas with regards to their housing conditions, access to basic services, socio-economic status and their psychosocial well-being. The survey included 1,057 individuals in randomly selected locations throughout the West Bank and Gaza. A total of 472 random interviews were also conducted in locations where people were at high risk of displacement, defined as Area C locations in the West Bank and locations near or adjacent to the Gaza buffer zone, a closed military area around the full perimeter of Gaza. The results showed the main reasons for displacement in west bank were Israeli orders. (31%), house demolition (23%) and inadequate shelter (15%). While in Gaza, the main factors behind displacement were house demolition (48%) and a lack of personal security (28%). Findings showed families that have been displaced fare significantly worse in terms of living conditions, socioeconomic impacts and psychosocial well-being than they did before their displacement, regardless of the reason why they were displaced. In addition, children of those families who have been displaced because of their demolished houses showed a decline in their mental health, suffering from traumas, sleeping problems, becoming withdrawn, depressed and anxious [47].

Al-Maqdese, is also one of the non-governmental organizations that have reported about consequences of home demolition has on the victims and also were responsible and dedicated to defend and protect the rights of Palestinians in East Jerusalem through carrying out face to face interviews and documenting the testimony of the people of East Jerusalem as well as legal support. The interviews were carried out with the assistance of a qualified psychologist, a field researcher and a translator who are all permanent members of Al-Maqdese staff. Al-Maqdese carries out regular workshops and counselling sessions with a large number of women affected by the housing demolitions in the city and as such the members of staff posess an in depth understanding of the situation of these women and the issues that most affect them. Al-Maqdese also offers psychological counselling for individuals adversely affected by housing demolitions in the city. For instance, all of the women to some extent, described the ongoing mental anxiety that results from the threat and experience of the loss of her home. Depression, anxiety, fear, and a lack of hope for the future were all common themes running through the interviews. Moreover, many women testified that not just her mental health but also her physical health has deteriorated as a result of the ongoing stress, fear, and anxiety associated with housing evictions, and the resultant displacement. According to Al-Maqdese Psychologist Zeinab Kaloti said, “Throughout my work I have worked with women suffering from the mental and physical effects of their home demolitions, some of them have been diagnosed with mental illness. Some suffered from Post-traumatic Stress Disorder (PTSD) showing symptoms such as anxiety, fainting, severe sweating and forgetfulness. Others suffered from Phobias, which presented themselves as extreme and abnormal fear when mentioning or seeing an Israeli soldier or member of the police force, this type of phobia is known as a ‘simple phobia’. Two women whom I met for treatment suffered from involuntary urination and one from Schizophrenia. One woman I treated suffered from Paranoid Schizophrenia which included the symptoms of suspicion, and auditory hallucinations while still others suffered from severe depression and anxiety” [31].

Another example of these NGO’s: Al Haq [48]. This NGO based in Ramallah calls themselves an independent Palestinian human right organization documents all types of human rights abuses in the Palestinian Territories and has several fieldworkers in the West bank. Palestinian families that have been a victim of human rights abuses can request their help. One thesis study was conducted in West Bank in 2015 and the researchers took the ability to access Al-Haq database and by using the search strings, respondents were chosen. Then, Al Haq field worker of the specific area called the respondents that were selected. The number of families who agreed to the interview in this study were distributed in different cities: Al–Far’aa refugee camp, south of Janin (1), Aida Refugee camp, near Bethlehem (1), Bethlehem (1), Hebron (3), Janin refugee camp (2), Jerusalem (2), Kufr Qaddum, between Nablus and Qalqilya (1), Nabulus (1), and Qalqilya (1). The families indicated that the Israeli forces appear mostly after midnight, to demolish the house. In all cases the soldiers closed and surrounded the area, not letting anyone in or out of the neighborhood. Soldiers entered the house without a warning and forced the family members out the house. In only a few cases, the family received a few minutes to collect their important belongings before their house was demolished. In most cases, the demolition was carried out by explosives combined with a bulldozer. Most participants explained that the home demolition made them anxious, and put under a high level of stress on the families. The period after the home demolition was perceived so stressful. It affected their daily lives, and ability to go to school or work or had concentration problems. Parents reported behavior changes and children deteriorated in school. For instance, children started sucking thumbs, wetting their beds or stopped allowing breastfeeding. Furthermore, children were sad, more nervous and scared [49].

On the other hand, there are some established studies that have been conducted to explore adolescent response to home demolitions. For instance, by using multi-group, cross-sectional study was conducted among adolescents in three groups in Bedouin villages, living in a recognized/legal village with no demolition, adolescents living in an unrecognized/ illegal village with no demolition, and adolescents living in an unrecognized/ illegal village with home demolition in order to examine how coping resources explain emotional reactions of anger and anxiety, in the context of threat of house demolition. The study was carried out during 2010–2011 and included 910 participants, of whom 411 adolescents lived in unrecognized villages where 193 of them experienced home demolition. Participants filled out a questionnaire including demographics, coping resources and emotional reactions. Results showed that adolescents from unrecognized villages whose homes have been demolished reported the highest stress reactions compared to the other groups. In addition, they reported significantly higher levels of state anger, state anxiety, and less hope compared to those whose homes were not demolished in an unrecognized villages and recognized villages. Those adolescents living in an unrecognized village with demolition, a negative correlation was found between hope and both anger and anxiety. The study concluded that demolition in the unrecognized villages is frequent, and due to the collective nature of these villages, adolescents, even if their own home was not demolished are in a state of continuous and intense exposure to the stressful situation of house demolitions [50].

Moreover, response/coping strategies such as receiving help from organizations, relying on social support networks and selling property are being used more widely in high-risk areas compared to the rest of the OPT. However, in high-risk areas, either coping strategies are either dwindling or not available [47]. A descriptive study was conducted in 2010 aimed to explore stress reactions of anxiety, anger and psychological distress as well as coping strategies among Bedouin Arab adolescents who were being exposed to the threat of house demolition in the unrecognized Bedouin villages in the Negev. The sample included four hundred and sixty-five Bedouin adolescents their age between 13 and 18 living in 19 unrecognized villages in southern Israel. No inclusion or exclusion criteria were used apart from age. Adolescents filled out self-report questionnaires, which included demographics, objective and subjective exposure to house demolition, state anxiety, state anger, psychological distress and Adolescent Coping Scale. Half of the sample reported their houses had been destroyed; a majority (92%) knew someone whose house was destroyed, and 82% knew someone who was physically hurt as a result of house demolition. Only 4% reported no exposure to any aspect of the five parameters of house demolition. Furthermore, adolescents reported their fear and danger of house demolitions for themselves, for their close family and extended family, for their friends and for people in their villages. Regarding coping, the study concluded that the nonproductive emotional coping strategies were positively linked to the psychological reactions for both groups, meaning that the greater the use of emotional coping, the more severe anxiety, anger and psychological distress [51].

During the interviews that carried within these studies, even more traumatic is the loss of so much that has personal and symbolic meaning. Photographs and personal papers, all dumped by a bulldozer into a damaged disreputable heap, alongside the stable necessities of everyday life–furniture, pots and pans, toilets, beds and toys. After a house is demolished, individuals must cope with the trauma in an environment of family trauma, which makes it much more difficult to receive the needed care. For children, who would normally be protected and cared for by their parents, the initial trauma is magnified. For instance, a young child who underwent a severe psychotic breakdown a few years after experiencing his home demolished several times [8]. Immediately after the demolition, most families are forced to find housing wherever they can, either crowding together or breaking up the family unit. A descriptive study was conducted in 2006 through collecting and analyzing stories reported by children in their everyday environments for example, their neighborhoods, schools and homes in the Gaza Strip. Six focus groups with 91 children aged 10–18 (48 female and 43 male) were conducted in Arabic by three local social workers who were known to the community. Their stories reveal that the demolitions of the family homes have been widespread and many families have found themselves on the streets with their children. They explained that the only safe place that provided shelter for a large number of displaced persons was school. When children were asked about the most painful incident that had happened to them, they all talked about losing their homes and becoming refugees in their own neighborhood. For them this internal displacement was perceived as a ‘double suffering’. Suffering from the effect of military occupation and its aggression that caused the loss of the home, and the social consequences of such loss that turned them into vulnerable individuals in their own societies. This was accompanied by feelings of subjugation, desperation and oppression. Twelve-year-old Suliman was very upset to have to live in the school, and said that “we want our schools to be for learning only, not for living in, just for education”. *While Samar, a 13-year-old girl, commented: ‘I felt the “qaher” [subjugation] and fear and was sad for our house’*. Nawal, a 13-year- old girl, stated that ‘we girls suffer more, girls without a home, is like being naked in public’. Another female child reported that female children perceived the loss of home as harder on women and girls. On the other hand, those younger children who had lost their homes reacted to their losses by trying to normalize their day-to-day activities. Many of them stated that directing their energies toward playing and helping others was a refuge and helped them to cope with their losses. Manal, a 14-year-old girl stated, while tears were welling up in her eyes: “I can’t even find words that could express to you how hard is it to lose your home, and see a bunch of stones, rubbles. But, seeing it this way is what motivated me to study, to help and support all friends and families that lost their homes” [52].

## Discussion

Home demolitions are a miserable and frequent reality of life for large part of Palestinian people. As a result, the social, economic, and psychological impact on individuals, families, and societies was clearly affected negatively [5]. In general, many theories have been suggested over the years to explain the developmental changes that people undergo over the course of their lives [53]. For example, the loss has been linked to symptoms of depression, the threat of anxiety, and frustration of aggression [54]. For Palestinians, the severe stress caused by losing a family home, and in fact the risk of its destruction, can’t only have just immediate, but long-term effects on the health of family members [31]. Moreover, threatened demolition and risk for displacement in the future might result in a situation in which "people live in constant fear and anxiety", with many essential items packed in case of demolition "happening suddenly, and they have to evacuate the home immediately." The probability of demolition itself, long before the soldiers arrived to destroy a house, is an affective form of violence against the residents of Palestine, as life under threat of demolition becomes loaded with fears and anxieties about the disturbing future and the violence that it carries, because such a policy of affective demolition actually works by making it easier to anticipate fear and anxiety [55]. It was clear from previous studies presented in Palestine that depression, anxiety, fear and lack of hope in the future were common themes running through interviews, for example, among women and their families in East Jerusalem and Arab Bedouin villages, especially since they are responsible for the struggle and protecting their children [31, 45]. In the Palestinian context, efforts to assert the right to home and place can be understood as an ongoing defense of the rights of residents to safety, identity, and well-being for not only themselves, but also for their children, families and communities [56]. Therefore, there is a pressing need to look around mental health condition for whom experienced their homes demolished along with those who have experienced a constant threat of demolition to their homes.

In general, parents, especially the mother, are the first to build a relationship with the child, thus they play the most important role in developing the child's mental and emotional characteristics and contribute crucially to the child's health or illness. It is well established that behavioral disorders among children reflect the psychological problems of parents, as the mental illness of either parent will increase the possibility of a child's mental disorder [57]. For example, a descriptive study showed that primary school children whose parents suffer from poor mental health also have more severe behavioral and emotional problems [58]. From previous studies presented in Palestine, it was clear that the period following the demolition of the home was very stressful for the parents and had a reflection on their children. For example, parents reported behavioral changes and children deteriorated in school. They also started sucking thumbs, wetting their beds or stopped allowing breastfeeding. Children were sad, more nervous and scared [49]. In general, a proper understanding of such a process and awareness of their own problems, along with developing parenting skills of coping can help parents reduce behavioral problems in children.

According to WHO, “The quality of housing has major implications for people’s health [59]. Research has shown that exposing to poor housing conditions, such as moisture, leakage and insufficient heating, increases the risk of respiratory infections and asthma, and to more serious conditions such as tuberculosis [60]. From previous studies presented in Palestine, families who were threatened with demolition, along with families who had been displaced, were living in poor housing conditions. These findings suggest a strong, long-term impact of persistent poor housing on health and mental health particularly. Studies have shown that people who live constantly in poor housing conditions today are more likely to develop poor housing conditions in the future. Moreover, the stress and anxiety of poor housing may last for a long time after the housing problems of individuals have improved [61]. Therefore, health services need to pay attention to these housing conditions, develop a clearer understanding and considering their implications throughout several aspects particularly mental health status as soon as possible.

Ordinarily, when a family has to move because of the home demolition, people lose their social network in their neighborhood. It was clear that suffering from the effect of military occupation and its aggression that caused the loss of the home, and the social consequences of such loss, turned them into vulnerable individuals in their own societies. When children were asked about the most painful incident that had happened to them, they all talked about losing their homes and becoming refugees in their own neighborhood [52]. Overall, neighbors in Palestine are seen as a fundamental cornerstone of society and neighborhoods consist of a lot of family members and being a ‘good’ neighbor is seen as an important duty [14, 14]. This may justify why younger children who had lost their homes reacted to their losses by trying to normalize their day-to-day activities by directing their energies toward helping others and helped them to cope with their losses [52]. Therefore, positive social support can play a crucial role in lowering psychological consequences. The social support for both families and neighborhoods helps individuals to cope with some of the problems [46].

Moreover, coping resources can be significant in reducing emotional responses because it helps in mediating the relationship between exposure to stress and psychological outcomes. Most models of coping assume that individuals who cope more effectively with stressful life events show lower levels of anxiety or depression [62]. Whereas several studies found that during acute violent situations (with political background) emotional coping strategies of denial and distancing were used more frequently [63]. It was clear in both groups of adolescents who participated in the study mentioned above [50, 51] that emotional coping strategies were linked to more anxiety, anger and psychological distress. Moreover, adolescents are at the stage of developing personal styles of coping. This developmental period also introduces abilities to deal with sources of conflict or stressful events in a variety of contexts [64]. Therefore, intervention and prevention programs should target facilitating coping strategies, which can help adolescents adjust in such a crisis.

Home demolition is a traumatic and difficult event for all the members of the family [65]. Interviewing traumatized people about traumatic events is difficult and might lead to additional problems [66]. For instance, respondents can endure extra stress because of retelling traumatic events. From the previous presented studies in Palestine, it was clear most of the researchers have used focus groups methods. It could be because these groups allow victims to share their suffering and tell their stories in their own environment while learning about each other’s ordeals, acknowledging their pains and hardships, sharing their own techniques of coping with loss, and even planning future supportive actions [52]. Those participants especially children who were silenced by fear and trauma may be able to speak up and share their ordeals, while being encouraged by the voices of the other children that spoke, wrote and resisted oppression even before any intervention. Therefore, as an interviewer, it is necessary to understand the impact of trauma on the brain, adjust the interview technique accordingly, and focus on empowering the interviewee. This would enhance their ability to recover from trauma.

Although many Palestinians have lost their security and stability after being demolished, more are still in place, competing on a daily basis with threats to the safety, quality and safety of their home environments [49]. Studies have shown value of both physical and psychological support in reducing the effects of war-related trauma, as well as the role of religion and cultural practices as methods of dealing with conflict situations [66]. Several factors seemed to lower the consequences of home demolition and seemed to work as a type of a coping mechanism among Palestinian population. Religion seemed to be a great supporter to cope with the consequences. For instance, Palestinians indicated that they would be rewarded and were able to find peace with their home demolition [50]. In addition, the faith and religious practices have positive effects for Islamic Palestinian population in reducing anxiety level such as praying and reading The Holly Quran. It enhances their resilience and ability to recover from continuous adversities and traumas. In addition to “Sumud Culture” which developed by Palestinians as a resilient response to the history of continuous traumas and threat to their existent on their homeland [67].

Overall, it is widely recognized that homes are critical sites for safety, meaning, belonging, and refuge, in addition to their important role in the formation and fortification of individual and collective identity and the nourishment of families and relationships [68]. As a result, losing one’s home can lead to a loss of identity, because the physical home is an important part of an individual’s identity [32]. As a result, home demolitions can alter, destroy Palestinians’ entire persona, and create different experience for each of men, women and children [36]. Personal identity must be protected under human rights law from deterioration and destruction. Because such of these inhuman and degrading handling affects a person's sense of identity. Violations the group's full identity through war crimes against humanity can kill lives, which necessarily means our individual identities. On a large context, it could lead to genocide or ethnic cleansing so that their identity no longer exists [69]. Therefore, there is a need to develop a comprehensive and coordinated response between human rights organizations and mental health services to prevent displacement and home demolition through providing a protection for those who have been displaced or at risk of displacement with a special focus on their specific needs and vulnerabilities [47].

In summary, it seems from above presented studies in Palestine that the psychological problems and mental consequences always follow the process of demolition or even the threat of demolition. Children, adolescents and women were a particularly vulnerable target group. Psychological problems were associated with different traumatic experiences and difficult events for all the members of the family. In some cases, trauma decrease children resilience since they became sad, more nervous and scared. Moreover, women may be particularly affected and significantly suffered of depression because they are a more vulnerable group in society for example: Arab Bedouin women, especially while they are working as the main caregivers in their families. Incidentally, while men are traditionally seen as family providers, there are no specific studies exploring their experience as husbands or parents and how they’ll cope. Therefore, to investigate the mental health of them, there is a need for drawing attention to the importance of providing an updated database in order to deal with several aspects of scientific research in Palestine and enhance the collaboration between all researchers.

## Conclusion

Depression, stress, anxiety, phobias and lack of hope in the future were mutual themes running throughout the lives of those families who have actually experienced home demolition, along with those who have experienced a constant threat of demolition to their homes, as well as many stressors of life such as lack of educational opportunities, low incomes, and a tendency to live in poor housing conditions after and before demolition also play a role in developing more mental disorders. Recommendations include ending the practice of home demolition in Palestine as a primary prevention. In addition, there is a great need to conduct a constant monitoring of the practice of home demolition in the OPT, the resulting displacement, the damage caused and ensure necessary assistance in particular mental health and psychological support for victims of home demolitions. Finally, there is a significant need to narrow down challenges facing mental health services in Palestine through placing a pressure on the government of Israel to end occupation that constitute violations of the human rights of Palestinians and thus threats to their mental health. We also recommend stopping support and arming Israel from USA and other European countries. This political pressure has a promise to stop the ethnic cleaning policy against Palestinians, promote justice and peace in the region. On the other hand, Palestinians need to enhance their resilience [70] and reduce the risk of developing mental illness such as PTSD, anxiety [71] and schizophrenia [72].

### Limitations

This paper has discussed home demolition in Palestine. Palestine is a state which seeks independence and freedom over the last 70 year [22]. Therefore, a mental health system is facing specific challenges linked with occupation and political conflict. Mental disorders remain underreported, under-resourced, under-treated, and mental health services underfunded [32]. Besides, the research approach is still underdeveloped. The results indicated a lack of established literature studies regarding the topic. Due to this shortage, reports that are related to the topic were included. Also, there is a lack of studies that discussed home demolition from mental health perspectives.

## Supplementary Information

**Additional file 1: Figure S1.** The map of historical Palestine.

**Additional file 2: Table S1.** Search history, **Table S2.** Studies Characteristics into two group Quantitative and Qualitative.

## Data Availability

This is an evidence synthesis study, all data is available from the primary research studies, or can be circulated from the corresponding author.
