# Supplementary material for: Mental health consequences of home demolition policy towered Palestinians: Literature review
Source: Int J Ment Health Syst. 2021 May 29;15:51. doi: 10.1186/s13033-021-00472-0 (PMC8164308; doi:10.1186/s13033-021-00472-0)
Supplement: Supplementary file 2 — Additional file 2: Table S1. Search history, Table S2. Studies Characteristics into two group Quantitative and Qualitative. [file 13033_2021_472_MOESM2_ESM.docx]

**Additional file 2:**

**Table S1. Search history**

| Comments |  | Number of hits | Search terms/keywords/ Combinations | Search # | Database/ search engine |
| --- | --- | --- | --- | --- | --- |
|  | 10 | 383,384 | Mental Health | 1 | PubMed  <https://pubmed.ncbi.nlm.nih.gov> |
|  |  | 3,085 | Palestine | 2 |  |
|  |  | 21 | Home demolition | 3 |  |
|  |  | 1 | Home demolition AND Palestine |  |  |
|  |  | 4 | Home demolition AND West bank |  |  |
|  |  | 2 | Home demolition AND Gaza Strip |  |  |
|  |  | 1 | Home demolition AND Mental Health |  |  |
|  | 14 | 209,000 | Home demolition | 1 | Google scholar  <https://scholar.google.com> / |
|  |  | 3,500,000 | Mental Health |  |  |
|  |  | 15,000 | Home demolition in Palestine |  |  |
|  |  | 52,300 | Home demolition AND Mental Health |  |  |
|  | 1 | 127 | Home demolition in Palestine | 1 | Science Direct  <https://www.sciencedirect.com> |
|  | 1 | 2,060 | Home demolition in Palestine | 1 | Semantic Scholar  <https://www.semanticscholar.org/> |
|  |  | 185 | Mental health | 1 | An-Najah University Journal for Research |

**Table S2. Studies Characteristics into two group Quantitative and Qualitative**

| Main Findings | Measurements | Methods | Aim | year | Author | Title of the study |
| --- | --- | --- | --- | --- | --- | --- |
| *Quantitative:* | | | | | | |
| Significantly more children exposed to bombardment and home demolition reported symptoms of post-traumatic stress (p=0·0008) and fear (p=0·002) than controls. | All children were assessed with the child post-traumatic stress reaction index (CPTSD-RI), the revised children’s manifest anxiety scale (RCMAS), and the children fears checklist. | 180 children: 91 (51%) who had been exposed to bombardment and home demolition, and 89 (49%) controls who had not. | To assess the nature and severity of emotional problems in Palestinian children whose homes had been bombarded and demolished during the crisis in Palestine, compared with children living in other parts of the Gaza strip. | 2002 | Thabet, A. A. M., Abed, Y., & Vostanis, P*.* | Emotional problems in Palestinian children living in a war zone: a cross-sectional study |
| 27.2% of participants reported that their house is under threat of demolition.  The Study concluded threat of housing demolition creates higher Depression Scale, even after adjusting for women’s, physical features of the house, and house location. | Depression Scale (CES-D) short form, which includes seven item-screening questions on DS experienced in the past week | A cross-sectional study conducted among 464 women who agreed to participate in the study and interviewed by using a structured Arabic-language questionnaire | To examine the relationship between risk of home demolition and symptoms of depression (DS) | 2014 | Daoud N, Jabareen Y. | Depressive Symptoms Among Arab Bedouin Women Whose Houses are Under Threat of Demolition in Southern Israel: A Right to Housing Issue |
| Adolescents from unrecognized villages whose homes have been demolished reported the highest stress reactions compared to the other groups. In addition, they reported significantly higher levels of state anger, state anxiety, and less hope compared to those whose homes were not demolished in an unrecognized villages and recognized villages. | via a questionnaire including demographics, coping resources and emotional reactions, The state anxiety index, The state anger index, Hope Questionnaire and Personal SOC were used. | using multi-group, cross-sectional carried out during 2010–2011 and included 910 participants, of whom 411 adolescents lived in unrecognized villages where 193 of them experienced home demolition | To investigate how coping resources, explain emotional reactions of anger and anxiety, in the context of threat of house demolition, among adolescents in ***three groups:*** Adolescents living in a recognized village with no demolition, adolescents living in an unrecognized village (by the Israeli government as legal) with no demolition, and adolescents living in an unrecognized village with demolition | 2018 | Al-Said H, Braun-Lewensohn O, Sagy S. | Sense of coherence, hope, and home demolition are differentially associated with anger and anxiety among Bedouin Arab adolescents in recognized and unrecognized villages |
| Half of the sample reported their houses had been destroyed. Nonproductive emotional coping strategies were positively linked to the psychological reactions for both groups, meaning that the greater the use of emotional coping, the more severe anxiety, anger and psychological distress | self-report questionnaires, which included demographics, objective and subjective exposure to house demolition, state anxiety, state anger, psychological distress and Adolescent Coping Scale. | The sample included four hundred and sixty-five Bedouin adolescents their age between 13–18 living in 19 unrecognized villages in southern Israel. | To explore stress reactions of anxiety, anger and psychological distress as well as coping strategies among Bedouin Arab adolescents who were being exposed to the threat of house demolition in the unrecognized Bedouin villages in the Negev. | 2013 | Al-Said H, Braun-Lewensohn O, Sagy S | Stress Reactions and Coping Strategies among Bedouin Arab Adolescents Exposed to Demolition of Houses |
| Main reasons for displacement in west bank were Israeli orders. (31%), house demolition (23%) and inadequate shelter (15%). While in Gaza, the main factors behind displacement were house demolition (48%) and a lack of personal security (28%).  families that have been displaced fare significantly worse in terms of living conditions, socioeconomic impacts and psychosocial well-being. | survey research methods using structured questionnaire | 1,057 individuals in randomly selected locations throughout the West Bank and Gaza. | To investigate the striking vulnerabilities of families living in high risk areas with regards to their housing conditions, access to basic services, socio-economic status and their psychosocial well-being. | 2009 | Save the Children | The struggle to survive and the impact of forced displacement in high-risk areas of the occupied Palestinian territory |
| *Qualitative:* | | | | | | |
| Depression, anxiety, fear, and a lack of hope for the future were all common themes running through the interviews. Moreover, many women testified that not just her mental health but also her physical health has deteriorated as a result of the ongoing stress, fear, and anxiety associated with housing evictions, and the resultant displacement. | Analyses of materials from a variety of  different sources. Codes and themes were used to classify, organize, and interpret data | Through carrying out face to face interviews and documenting the testimony of the people of East Jerusalem as well as legal support. | To continue to create a comprehensive data bank containing details of housing demolitions that have taken place in the city from 1967 to the present day. | 2011 | Human Rights First | The loss of homes and hope. The effects of housing demolitions and forced evictions on the rights of Women in East Jerusalem and an assessment of the applicable legal framework |
| In most cases, the demolition was carried out by explosives combined with a bulldozer. Most participants explained that the home demolition made them anxious, and put under a high level of stress on the families. The period after the home demolition was perceived so stressful. It affected their daily lives, and ability to go to school or work or had concentration problems. Children started sucking thumbs, wetting their beds or stopped allowing breastfeeding. Furthermore, children were sad, more nervous and scared. | In-depth semi – structured interviews. Codes and themes were used to classify, organize, and interpret data | **Twelve families agreed** to the interview and distributed in different cities in Westbank. | To investigate what punitive home demolitions are, how these are carried out  and what the consequences for the uninvolved inhabitants of the houses are. | 2015 | Van Der Meijden A, Stuifbergen J. | The consequences of punitive home demolitions in the occupied Palestinian territories |
| Suffering from the effect of military occupation and its aggression that caused the loss of the home, and the social consequences of such loss that turned them into vulnerable individuals in their own societies. This was accompanied by feelings of subjugation, desperation and oppression. | Listening to, and systematically reporting on their stories. | A descriptive study was conducted through collecting and analyzing stories reported by children by using Six focus groups with 91 children aged 10–18 (48 female and 43 male) | To capture children’s complex understandings of, and responses to, the trauma of ongoing militarization and political occupation as reflected in their perceptions of the loss of their homes | 2006 | Shalhoub-Kevorkian N. | The Political Economy of Children’s Trauma: A Case Study of House Demolition in Palestine |
| Women's narratives of relentless surveillance highlight their constant doubt regarding the integrity of their homes. One woman said simply, “This is our life,” and other said, “Where is our freedom?” | Codes and themes were used to classify, organize, and interpret data | Focus groups of 32 adult Palestinian women in different sectors of the West Bank. | To explore the implications of violations to the home within political violence. | 2008 | Sousa CA, Kemp S, El-Zuhairi M. | Dwelling within political violence: Palestinian women’s narratives of home, mental health, and resilience. |
